# Shorter Leukocyte Telomere Length coupled with lower expression of Telomerase Genes in patients with Essential Hypertension

**DOI:** 10.7150/ijms.48456

**Published:** 2020-08-01

**Authors:** Guanghui Cheng, Lina Wang, Mingkai Dai, Fengtao Wei, Dawei Xu

**Affiliations:** 1 Central Research Laboratory, The Second Hospital, Cheeloo College of Medicine, Shandong University, Jinan, 250033, PR China.; 2 Department of Cardiology, The Second Hospital, Cheeloo College of Medicine, Shandong University, Jinan, 250033, PR China.; 3 Department of Medicine, Division of Hematology, Bioclinicum and Center for Molecular Medicine, Karolinska Institutet and Karolinska University Hospital Solna, SE-171 76 Solna, Sweden.

**Keywords:** Age-related disease, Hypertension, ET-1, Telomerase, Telomere

## Abstract

**Background:** The essential hypertension (EH) pathophysiology remains poorly understood. Many studies indicate that reduced leukocyte telomere length (LTL) is involved in the EH pathogenesis, however, the direct analysis of arterial telomere length (ATL) from EH patients and normotensive individuals did not show a difference. To address these discrepant observations between LTL and ATL, we performed comprehensive analyses of LTL, telomerase gene expression and their genetic variants in healthy normotensive controls and EH patients.

**Methods:** Sex-matched 206 EH patients and equal numbers of healthy controls were recruited. LTL, and the expression of two key telomerase components, telomerase reverse transcriptase (TERT) and internal RNA template (TERC) were determined using qPCR. Genetic variants of rs2736100 at the *TERT* and rs12696304 at the *TERC* loci were determined using TaqMan genotyping kits.

**Results:** LTL was significantly shorter in EH patients than in their normotensive controls (0.96 ± 0.52 vs 1.19 ± 0.58, *P* = 0.001). Moreover, TERT and TERC expression in patients' leukocytes were substantially lower compare to that in healthy controls (TERT, 0.98 ± 0.98 vs 1.76 ± 1.75, *P* = 0.003; TERC, 1.26 ± 1.62 vs 4.69 ± 3.61, *P* < 0.001). However, there were no differences in the genetic variants of rs2736100 and rs12696304 between patient and control groups.

**Conclusions:** EH patients have significantly shorter LTL, which may result from defective TERT and TERC expression in leukocytes. Collectively, lower telomerase expression contributes to shorter LTL observed in EH patients, and telomerase activators may be considered for EH therapy.

## Introduction

Human linear chromosomes terminate with 8-20 kb long TTAGGG repeat sequences, and these DNA repeats together with its binding factors form ribonucleoprotein structures so-called telomeres 1-3. Such telomere structures form protective cap on chromosomes to maintain genomic stability and integrity. Telomeric DNA or TTAGGG repeats are synthesized by an RNA dependent DNA polymerase named telomerase, a multi-unit enzyme complex with telomerase reverse transcriptase (TERT) and telomerase RNA template (TERC) as its core components 1, 2. Normal human stem cells, lymphocytes and other highly proliferative cells contain telomerase activity, but most human somatic cells express negligible or low levels of telomerase activity, and undergo progressive telomere shortening with cellular divisions due to “the end-replication problem” 1, 3, 4. When telomeres become too short to protect chromosomes, unprotected chromosome ends mimic the doubled strand DNA breaks, and thereby activate the DNA damage response, which consequently trigger replicative senescence, a permanent growth arrest status 1, 3. Thus, telomere shortening acts as a mitotic clock to count the number of cell divisions and to limit cellular life-span. The same scenario also occurs *in vivo* with increased age, and therefore shortened telomeres are widely accepted as a biomarker for aging and age-related conditions 1, 3, 5. Indeed, by assessing leukocyte telomere length (LTL), many studies have shown that shorter LTL is associated with increased mortality, and ag-related diseases including cancer, cardiovascular disorders, stroke, diabetes, and among others 3, 6. In addition, certain genetic variants or single nucleotide polymorphisms (SNPs) at *TERT* and *TERC* genes, for instance, TERT rs2736100 and TERC rs12696304 have been shown to significantly impact telomere length by either up-regulating telomerase or other mechanisms, and in turn contribute to age-related disorders 7-18.

Hypertension is a common age-related disease that causes atherosclerosis, stroke, and chronic kidney disease, and associates closely with morbidity and mortality worldwide 19, 20. In up to 95% of patients with hypertension, exact driving-factors are unknown, so-called essential hypertension (EH) 21. It is in general thought that genetic and environmental elements interact to result in EH, and in this pathogenic process, age-related changes may play a pivotal role 19, 20, 22. Indeed, vasculature dysfunction, inflammation and oxidative stress are common mechanisms promoting both biological aging and EH development 19, 20, 23-25. It has been shown that the senescence of vascular smooth muscle and endothelial cells resulting from telomere erosion with cellular replication or increased age is a key driver for vasculature dysfunction 21, 26, 27. Matsushita et al. observed that human senescent endothelial cells produced significantly lower levels of the vasodilator nitric oxide (NO), while ectopic TERT expression in these cells restored the NO production 28. In TERC-knockout mice at third generation, shortened telomeres induced higher plasma endothelin-1 (ET-1), a potent vasoconstrictor 29. Conceivably, decreased NO and/or increased ET-1 contributes to hypertension development. In accordance with the *in vitro* experiments and mouse studies above, several studies showed that patients with EH had shorter LTL compared with that in healthy normotensive individuals 25, 30-35. Unexpectedly, however, the direct analysis of arterial telomere length (ATL) from EH patients and normotensive individuals did not show a difference, which suggests that LTL may not necessarily reflect changes in ATL in EH patients 36. It is thus important to elucidate the mechanism(s) underlying shorter LTL in EH. Because telomerase is the only enzyme to maintain telomere length in lymphocytes (the predominant cell type in peripheral leukocytes) 2, 37, 38, we simultaneously determined LTL and telomerase gene expression in normotensive controls and EH patients to address the above issue. In the meanwhile, we further compared the genetic variants of rs2736100 and rs12696304, two SNPs genetically associated with telomere length, between patients and controls.

## Subjects and Methods

### Study populations

The study includes 206 patients with EH recruited from Shandong University Second Hospital between Jan. 2018 and June 2019. The EH diagnosis was made according to the diagnostic criteria 2010 Chinese guidelines for the management of hypertension (>140 systolic mmHg and >90 diastolic mmHg). Two-hundreds and twenty-six unrelated normotensive healthy individuals as controls were from the Physical Examination Center of Shandong University Second Hospital. The healthy controls and patients were sex-matched with age differences within 5 years old, and they were all Han Chinese. The study was approved by the Ethics Review Committee of Shandong University Second Hospital and informed consent was obtained from all participants.

### DNA extraction and LTL assay

Genomic DNA was extracted from peripheral blood cells using TIANGEN DNA extraction kits (Tiangen Biotech, Beijing, China). LTL was assessed using real-time PCR as previously described 13, 39. Briefly, 2 ng of DNA were used for each PCR reaction. The primer sequences for human telomere (Tel 1b and Tel 2b) and β-globin (HBG3 and HBG4) were: Tel1b: 5'-CGGTTTGTTTGGGTTTGGGT-TTGGGTTTGGGTTTGGGTT-3'; Tel2b: 5'-GGCTTGCCTTACCCTTACCCTTACCC-TTACCCTTACCCT-3'; HBG3: 5'-TGTGCTGGCCCATCACTTTG-3', and HBG4: 5'-ACCAGCCA-CCACTTTCTGATAGG-3'. T/HBG (S) values (T: telomere repeat copy number, and S: single copy gene number) were determined using the formula T/S = 2-ΔCt, where ΔCt = average Ct_telomere_ - average Ct_β-globin_. The T/S ratio was arbitrarily expressed as LTL. Age-adjusted LTL for each control and patient was done by subtracting the subject's linear predicted LTL from the observed one.

### RNA extraction and quantitative polymerase chain reaction (qPCR)

Blood was drawn from both healthy controls and EH patients and mononuclear cells were then isolated by centrifugation with Ficoll-Paque (GE Healthcare, China). RNA was extracted using Trizol (Life technologies, Carlsbad, CA) according to the manufacturer's protocol. For reverse transcription, High-Capacity cDNA Reverse Transcription Kit (Applied Biosystems, Waltham, MA) was used. Real time amplification was performed in triplicate using SYBR Green PCR Master Mix (Life Technologies, Carlsbad, CA) with QuantStudio 5 Flex Teal-Time PCR system (Applied Biosystems, Waltham, MA). The primers were purchased from Invitrogen (Waltham, MA) and their sequences are as follow: TERT, 5'-CGG AAG AGT GTC TGG AGC AA-3' (Forward) and 5'-GGA TGA AGC GGA GTC TGG A-3´ (Reverse); TERC, 5´-TCA GCG TCA GCC TCC TCT T-3' (Forward) and 5'-TTG ATG TCC GCC AGG TTG AA-3' (Reverse); β2-M, 5'-GAA TTG CTA TGT GTC TGG GT-3' (Forward) and 5'-CAT CTT CAA ACC TCC ATG ATG-3' (Reverse). Levels of TERT and TERC mRNA were arbitrarily expressed as the ratio of the target/β2-M according to the protocol from QuantStudio 5 Flex. In all the assays, positive (bladder cancer cell line) and negative controls (H2O) were included. For positive controls, cDNA samples were diluted at 0-, 10-, 100- and 1000-fold, respectively, to make a standard curve. CT values in PBMC samples are all within arranges of standard curves. RNA samples without a reverse transcription step were also included as additional controls for potential genomic DNA contamination.

### Genotyping of the TERT rs2736100 and TERC rs12696304 variants

The *TERC* rs12696304(C/G) and *TERT* rs2736100 (A/C) genotyping was carried out using pre-designed TaqMan SNP genotyping assay kits on an ABI 7500 Life Tech (Applied Biosystems), as described 8, 13. Both positive and negative controls were included in all assays and PCR was run using the following conditions: 95°C for 5 min, followed by 40 cycles of 92°C for 15 s and 60°C for 30 s.

### Plasma ET-1 assessment

Plasma was collected from both healthy controls and patients and ET-1 levels were assessed using an ET-1 ELISA kit (Elabscience^®^, Wuhan, China) according to the manufacture's protocol. Briefly, 100 μL of plasma were added into the ELISA plate pre-coated with an ET- antibody and incubated at 37°C for 90 mins. HRP-conjugated monoclonal antibody was then added for incubation and washed, followed by the addition of substrates. Optical density was finally determined at 450 nm using an ELISA reader. The concentration of plasma ET-1 (pg/ml) was calculated according to the standard curve included in assays.

### Statistical analyses

The difference in LTL, TERT, TERC, and ET-1 between patients and healthy controls was assessed using Student T-test. The evaluation of distribution differences in alleles of the *TERC* rs12696304 and *TERT* rs2736100 between patients and healthy controls were done using χ^2^ test. Unconditional univariate and multivariate logistic regression analyses were used to estimate Odd ratios (ORs) for risk of EH and their 95% confidence intervals (CIs). Spearman's rank correlation coefficient was applied to determine the relationship between ET-1 and LTL, TERT or TERC. The relationship between LTL and age was assessed by Pearson's test. All the tests were computed using SPSS17.0 software. *P* values of < 0.05 were considered as statistically significant.

## Results

### Characteristics of the study participants

A total of 206 patients with EH were included in the present study, and their age and sex were listed in Table 1. One or more prescribed medications were used to treat patients' blood pressure. The same number of unrelated normotensive healthy adults used as controls were sex-matched with age difference (SD) <5years old (Table 1). Both controls and patients were analyzed for blood cholesterol, triglycerides, and low and high density liopoproteins. EH patients had significantly higher levels of low density liopoproteins, while reduced high density liopoproteins and cholesterol (Table 1).

### Shorter LTL and lower TERT and TERC expression in patients with EH

**Figure 1:**
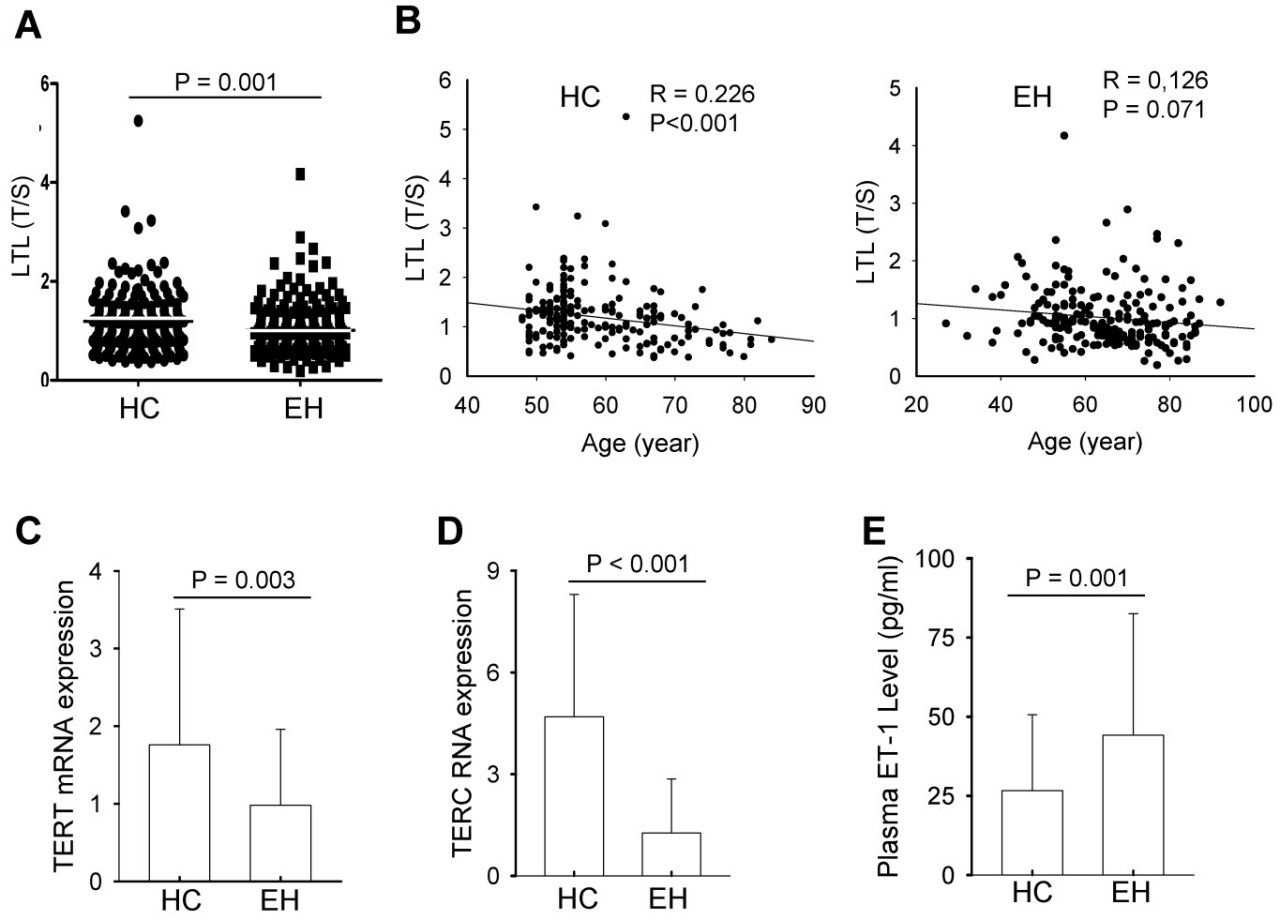
** Leukocyte telomere length (LTL), telomerase expression and plasma ET-1 levels in healthy controls (HCs) and patients with essential hypertension (EH).** (**A**) Shorter LTL in EH patients. Genomic DNA extracted from peripheral blood was assesses for telomere length using qPCR and the ratio of telomere signals to single copy gene signals (T/S) arbitrarily expressed as relative telomere length. (**B**) The negative correlation between LTL and age in HCs (Left panel) and EH patients (Right panel). (**C and D**) TERT and TERC expression, respectively in HCs and EH patients. Total RNA was isolated from PBMCs from HCs and EH patients and qPCR was applied for the analysis of TERT and TERC RNA levels. (**E**) Plasma ET-1 levels in HCs and EH patients. The quantification was performed using ELISA.

Genomic DNA extracted from peripheral leukocytes was used to assess telomere length with qPCR. LTL showed a wide variation in individuals from both control and patient groups, and average length after the adjustment of age difference was 1.19 ± 0.58 and 0.96 ± 0.52 (mean ± SD) for normotensive controls and EH patients, respectively (Fig. 1A). The difference was highly significant (*P* = 0.001). A significant correlation between LTL and age was observed in the control group (R = 0.226, *P*<0.001), while LTL and age tended to be correlated with each other, but the statistical significance was not achieved (at a border line) (R = 0.126, *P* = 0.071) (Fig. 1B).

To determine whether shorter LTL in patients is due to insufficient telomerase activity, we isolated peripheral blood mononuclear cells (PBMCs) from both controls and patients, and analyzed TERT and TERC expression using qPCR. The levels of TERT mRNA varied widely, but EH patients showed significantly lower abundances of TERT mRNA in their PBMCs compared with those in controls' PBMCs (0.98 ± 0.98 vs 1.76 ± 1.75, *P* = 0.003) (Fig. 1C). Similarly, TERC RNA expression fluctuated markedly among normotensive controls and EH cases. The TERC level in BPMCs derived from normotensive controls was more than 3-fold higher than that in EH patients, and the difference was highly significant (1.26 ± 1.62 vs 4.69 ± 3.61, *P* <0.001) (Fig. 1D).

### Lack of differences in rs2736100 and rs12696304 genotypes between EH patients and healthy controls

Given the findings above, we further sought to explore potential mechanisms underlying down-regulation of TERT and TERC expression in EH. It is known that the SNPs rs2736100 at *TERT* and rs12696304 at *TERC* are genetically involved in the regulation of LTL and gene expression 8, 9, 11-13. Thus, we analyzed the genotype distribution of these two SNPs in normotensive controls and EH patients. Successful genotyping was achieved in 203 and 204 patients for rs2736100 and rs12696304, respectively. As shown in Table 2, the rs2736100 genotyping showed no differences in AA, AC and CC variants between controls and patients. The lack of differences in the variants of rs12696304 was similarly observed (Table 2). Because the variants at these two SNPs are differently associated with disease susceptibility between males and females 8, 13, 18, we further compared their distributions among males and females separately. There were no differences between controls and patients either males or females (Table 3 and Table 4).

### Increased plasma ET-1 levels in patients with EH independently of LTL and TERT/TERC expression

In late generations of TERC-knockout mice, critically shortened telomeres lead to increased ET-1 through which hypertension is induced 29. We thus wanted to determine whether ET-1 increased in EH patients and whether the plasma level of ET-1 was associated with LTL or TERT/TERC expression. As expected, EH patients had significantly higher plasma ET-1 than did normotensive controls (44.14 ± 38.36 pg/ml vs 26.65 ± 23.86 pg/ml, *P* = 0.001) (Fig. 1E). However, neither in the control group nor patient group, the correlation of ET-1 levels with LTL, TERT or TERC expression was observed (Normotensive controls: ET-1 vs LTL, TERT and TERC, ρ = -0.096, *P* = 0.445, ρ = -0.034, *P* = 0.780, and ρ = -0.030, *P* = 0.800, respectively; EH patients: ρ = -0.082, *P* = 0.469, ρ = 0.200, *P* = 0.139, and ρ = -0.054, *P* = 0.669, respectively).

## Discussion

In the present study, we comprehensively analyzed LTL, TERT and TERC expression and their genetic variants in patients with EH. Significantly shorter telomeres coupled with lower levels of TERT and TERC expression were observed in leukocytes derived from patients with EH. The findings are unexpected, and promote us to re-consider the relationship between shorter LTL and EH.

It is known that LTL is affected by environmental factors or lifestyles including smoking, alcohol abuse, obesity, psychological stress, lack of exercise and others 3, while all these elements are also associated with increased risk of EH and other cardiovascular diseases 21, 25. Therefore, it is difficult to distinguish whether shorter LTL and EH are only a coincidence or causally related. Moreover, the direct analysis of arterial telomere length (ATL) from EH patients and normotensive individuals did not show a difference 36, which suggests that shorter LTL does not necessarily reflect the same alteration in ATL in EH patients. To address these different scenarios, we determined the expression of TERT and TERC, two essential components constituting the core of the telomerase enzyme complex, in peripheral blood mononuclear cells where lymphocytes are predominant cell types. Normal human lymphocytes express basal levels of TERT mRNA and TERC RNA, and as expected, their transcripts were readily detectable in blood cells derived from both healthy controls and EH patients. However, compared to those in healthy controls, patients' cells expressed significantly lower abundances of TERC and TERT mRNA. These findings indicate that TERC and TERT expression is defective in patients' blood cells, which in turn leads to the reduced LTL. Consistently, Tedone et al. recently showed that longer telomere length and high telomerase activity in peripheral T lymphocytes are useful biomarkers for centenarians who lacked age-related diseases 40.

TERT and TERC expression in PBMCs derived from both normotensives and hypertensives exhibits large variations, and one potential explanation is the difference in cell sub-populations in PBMCs. It is well-known that TERT is predominantly expressed in T lymphocytes, while the proportion of T cells varies from one to one. However, TERC is ubiquitously present in all PBMCs, and its expression variation is unlikely due to different cell compositions. Nevertheless, a significant down-regulation of TERT and TERC expression is observed in EH patients. Because telomere length and telomerase expression is controlled genetically and associated with certain genetic variants of the TERT and TERC loci 1, we further determined the genetic variants at TERT rs2736100 and TERC rs12696304, two well studied SNPs affecting telomere length and/or telomerase expression 8, 9, 11-13, 18. However, there were no differences in the genotype of these two SNPs between normotensive healthy controls and EH patients, indicating that other unknown factors contribute to reduced LTL and TERT/TERC expression observed in EH here. It should be pointed out that the analyzed cohorts are not big enough to make a solid conclusion. Further investigations are required to clarify this issue. Intriguingly, our recent finding showed that these two SNPs were significantly associated with susceptibility of primary glomerulonephritis and chronic kidney disease 18, the major contributors to secondary hypertension.

A similar scenario occurs in the autoimmune disease rheumatoid arthritis (RA), too. Several early studies showed that RA patients had significantly shortened LTL 41, which was thought that the accelerated telomere attrition participated in the pathogenesis of RA. However, Fujii et al. observed that TERT expression was defective in naive T cells from RA patients upon their stimulation, which consequently led to impaired telomere maintenance, poor proliferation and lymphopenia 42. When defective telomerase activity was corrected, immune abnormalities were improved in RA 42. EH has been proposed as an autoimmune disease and inflammation plays a pivotal role, and it is worth dissecting whether the same mechanism is involved in the EH development.

PBMCs or T lymphocytes serve as peripheral biomarkers mirroring alterations within a multitude of organs and tissues 40 and a useful cell model to study human aging. However, it is currently unclear whether reduced expression of TERT and TERC observed in PBMCs occurs in endothelial cells from EH patients, too. Imanishi et al reported that bone marrow endothelial cells derived from both hypertensive rat models and EH patients had lower levels of telomerase activity and shorter telomere length, but the analyses were performed after *in vitro* culture, which did not necessarily reflect an *in situ* situation 26. In a recent study, however, the accelerated attrition of ATL was not documented by directly determining telomere length in primary aortic tissues derived from EH patients 36, which seems not to support the insufficient TERT/TERC expression. In addition, we observed significantly higher plasma levels of ET-1, but its abundances were related to neither LTL, nor TERT and TERC expression. These findings indicate that other mechanisms rather than shortened LTL contribute to increased ET-1 in EH patients. A thorough analysis of telomere length and TERT/TERC expression in *in vivo* endothelial cells from EH patients are required to delineate this issue. If telomerase gene expression is down-regulated in EH endothelial cells, a telomerase activator TA65, which up-regulates TERT expression 43, may be tested as an anti-hypertensive treatment.

In summary, the findings presented herein show a significantly lower expression of TERT and TERC in PBMCs or lymphocytes derived from EH patients, which may provide an explanation for shortened LTL observed in EH. The down-regulation of TERT and TERC expression is not associated with the genetic variants at rs2736100 or rs12696304, and the exact underlying mechanism is currently unclear, which calls for further investigations. More importantly, it remains to be defined whether the finding from PBMCs mirrors the changes in endothelial cells. If this is the case, anti-aging telomerase activators, such as TA65 or others 43, may be tested as a therapeutic strategy for EH. Collectively, the present findings indicate the role for telomerase insufficiency in the EH pathogenesis and may be implicated in the EH intervention.

## Figures and Tables

**Table 1:** The summary of characteristics in healthy normotensive controls and EH patients

	Control (N)	Case (N)	*P* value
N (Total )	206	206	
Age (years)	59 ± 8	64 ±12	
Gender (male/female)	100/106	100/106	
Total cholesterol	4.96 ± 1.02	4.26 ± 1.15	<0.001
Triglycerides	1.18 ± 0.51	1.35 ± 0.96	0.210
Low density lipoprotein	2.22 ± 0.60	2.89 ± 1.59	0.001
High density lipoprotein	1.81 ± 0.31	1.23 ± 0.48	<0.001

EH: Essential hypertension.

**Table 2:** The summary of rs2736100 and rs12696304 genotyping in healthy controls and EH patients

Genotypes	Control	Case	Odds ratio (95% CI)	*P* value
rs2736100 (N)	203 (100%)	203 (100%)		
AA	70 (34.5)	61 (30.0)	1.0 (ref.)	
AC	97 (47.8)	107 (52.7)	0.790 (0.509 - 1.226)	0.293
CC	36 (17.7)	35 (17.3)	0.896 (0.503- 1.598)	0.711
AC + CC	133	142	0.816 (0.538 - 1.238)	0.339
A	237 (58.4)	229 (56.4)		
C	169 (41.6)	177 (43.6)	1.084 (0.821 - 1.432)	0.570
**rs12696304 (N)**	**203 (100%)**	**204 (100%)**		
GG	102 (50.2)	102 (50.0)	1.0 (ref.)	
GC	80 (39.4)	83 (40.7)	0.964 (0.638 - 1.455)	0.861
CC	21 (10.4)	19 (9.3)	1.105 (0.561- 2.179)	0.772
GC + CC	101	102	0.990 (0.671 - 1.460)	0.960
G	284 (70.0)	287 (70.3)		
C	122 (30.0)	121 (29.7)	0.981 (0.727 - 1.325)	0.903

EH, Essential hypertension; CI, Confidence interval.

**Table 3:** The rs2736100 genotyping in male and female HCs and EH patients

	Male			
Genotype	HC	EH	Odds ratio (95% CI)	*P* value
N	98 (100%)	99 (100%)		
AA	30 (30.6)	29 (29.3)	1.0 (ref.)	
AC	48 (49.0)	53 (53.5)	0.875 (0.460 - 1.655)	0.685
CC	20 (20.4)	17 (17.2)	1.137 (0.499 - 2.592)	0.760
AC + CC	68	70	0.939 (0.510 - 1.728)	0.840
A	108 (55.1)	111 (56.1)		
C	88 (44.9)	87 (43.9)	0.962 (0.646 - 1.431)	0.848
	**Female**			
**Genotype**	**HC**	**EH**	**Odds ratio (95% CI)**	***P* value**
N	105 (100%)	104 (100%)		
AA	40 (38.1)	32 (30.8)	1.0 (ref.)	
AC	49 (46.7)	54 (51.9)	0.726 (0.397 - 1.329)	0.299
CC	16 (15.2)	18 (17.3)	0.711 (0.314 - 1.612)	0.413
AC + CC	65	72	0.722 (0.407 - 1.281)	0.265
A	129 (61.4)	118 (56.7)		
C	81 (38.6)	90 (43.3)	1.215 (0.822 - 1.795)	0.329

CI, Confident interval; Ref., Reference; HC, Healthy controls; EH: Essential hypertension.

**Table 4:** The rs12696304 genotyping in male and female HCs and EH patients

	Male			
Genotype	HC	EH	Odds ratio (95% CI)	P value
N	100 (100%)	98 (100%)		
GG	52 (52.0)	51 (52.0)	1.0 (ref.)	
GC	36 (36.0)	37 (37.8)	0.954 (0.524 - 1.738)	0.878
CC	12 (12.0)	10 (10.2)	1.177 (0.467 - 2.965)	0.729
GC + CC	48	47	1.002 (0.573 - 1.749)	0.995
G	140 (70.0)	139 (70.9)		
C	60 (30.0)	57 (29.1)	0.957 (0.621 - 1.474)	0.841
	**Female**			
**Genotype**	**HC**	**EH**	**Odds ratio (95% CI)**	***P* value**
N	103 (100%)	106 (100%)		
GG	50 (48.6)	51 (48.1)	1.0 (ref.)	
GC	44 (42.7)	46 (43.4)	0.976 (0.553 - 1.722)	0.932
CC	9 (8.7)	9(8.5)	1.020 (0.374 - 2.781)	0.969
GC + CC	53	55	0.983 (0.571 - 1.691)	0.950
G	144(69.9)	148(69.8)		
C	62(30.1)	64(30.2)	1.004 (0.661 - 1.525)	0.984

CI, Confident interval; Ref., Reference; HC, Healthy controls; EH: Essential hypertension.

## Abbreviations

ATL: Arterial telomere length
CI: Confidence intervals
EC: Endothelial cell
EH: Essential hypertension
ET-1: Endothelin-1
LTL: Leukocyte telomere length
NO: Nitric oxide
OD: Odd ratio
PBMCs: Peripheral blood mononuclear cells
SNP: Single nucleotide polymorphisms
TERC: Telomerase RNA template
TERT: Telomerase reverse transcriptase
